# Omega-3 Polyunsaturated Fatty Acids Antagonize Macrophage Inflammation via Activation of AMPK/SIRT1 Pathway

**DOI:** 10.1371/journal.pone.0045990

**Published:** 2012-10-05

**Authors:** Bingzhong Xue, Zhenggang Yang, Xianfeng Wang, Hang Shi

**Affiliations:** 1 Department of Biology, Georgia State University, Atlanta, Georgia, United States of America; 2 Department of Internal Medicine, Wake Forest University School of Medicine, Medical Center Boulevard, Winston-Salem, North Carolina, United States of America; Warren Alpert Medical School of Brown University, United States of America

## Abstract

Macrophages play a key role in obesity-induced inflammation. Omega-3 polyunsaturated fatty acids (ω-3 PUFAs) eicosapentaenoic acid (EPA) and docosahexaenoic acid (DHA) exert anti-inflammatory functions in both humans and animal models, but the exact cellular signals mediating the beneficial effects are not completely understood. We previously found that two nutrient sensors AMP-activated protein kinase (AMPK) and SIRT1 interact to regulate macrophage inflammation. Here we aim to determine whether ω-3 PUFAs antagonize macrophage inflammation via activation of AMPK/SIRT1 pathway. Treatment of ω-3 PUFAs suppresses lipopolysaccharide (LPS)-induced cytokine expression in macrophages. Luciferase reporter assays, electrophoretic mobility shift assays (EMSA) and Chromatin immunoprecipitation (ChIP) assays show that treatment of macrophages with ω-3 PUFAs significantly inhibits LPS-induced NF-κB signaling. Interestingly, DHA also increases expression, phosphorylation and activity of the major isoform α1AMPK, which further leads to SIRT1 over-expression. More importantly, DHA mimics the effect of SIRT1 on deacetylation of the NF-κB subunit p65, and the ability of DHA to deacetylate p65 and inhibit its signaling and downstream cytokine expression require SIRT1. In conclusion, ω-3 PUFAs negatively regulate macrophage inflammation by deacetylating NF-κB, which acts through activation of AMPK/SIRT1 pathway. Our study defines AMPK/SIRT1 as a novel cellular mediator for the anti-inflammatory effects of ω-3 PUFAs.

## Introduction

Chronic inflammation has emerged as one of the key physiological mechanism linking obesity to insulin resistance/type 2 diabetes [1]. Obesity-associated chronic inflammation features increased production of pro-inflammatory cytokines and activation of the inflammatory pathways in key metabolic tissues [1]. It is increasingly recognized that adipose tissue plays a key role in obesity-induced inflammation [1]. Further studies provided solid evidence that adipose tissue in obesity displays increased infiltration of macrophages, and that a major source of the adipose inflammation comes from infiltrated macrophages [2], [3]. The role of macrophages in obesity-induced inflammation and insulin resistance has been extensively investigated in a number of genetic models [4], [5], [6], [7]. For instance, targeted deletion of IKK-β in myeloid lineage cells protected mice from high-fat (HF) diet-induced inflammation and insulin resistance [4]. Similarly, JNK1 deletion in hematopoietic cells including macrophages also ameliorated obesity-induced inflammation and insulin resistance in mice [5]. In contrast, myeloid specific deletion of peroxisome proliferator activated receptor-γ (PPAR-γ) increased systemic inflammation and impaired insulin sensitivity in mice [6], [7]. These genetic studies demonstrate that altered macrophage inflammation plays a critical role in obesity-induced inflammation and thereby leads to systemic insulin resistance in obesity. Therefore, searching for novel agents that can antagonize macrophage inflammation may represent a therapeutic strategy for the prevention and treatment of insulin resistance and type 2 diabetes.

ω-3 polyunsaturated fatty acids (ω-3 PUFAs) have shown potent anti-inflammatory effects in disease models featuring chronic inflammation [8], [9](see reviews [10], [11], [12]). The mechanisms underlying ω-3 PUFAs' anti-inflammatory functions have received investigation. Several plausible theories have been advanced to explain the ability of ω-3 PUFAs to antagonize inflammation and include competitive inhibition of conversion of arachidonate to pro-inflammatory lipid intermediates, serving as endogenous ligands for PPARγ, generation of anti-inflammatory lipid mediators such as resolvins and protectins, and activation of GPR120 [11], [13], [14], [15], [16], [17], [18]. However, the cellular signals mediating ω-3 PUFAs' anti-inflammatory effects are not completely understood.

We previously found that two nutrient sensors AMP-activated protein kinase (AMPK) and SIRT1 interact to regulate macrophage inflammation [19]. Indeed, AMPK activation deacetylates NF-κB, which acts through SIRT1, and therefore leads to inhibition of NF-κB signaling and cytokine expression [19]. Our observations raise an interesting question as to whether the anti-inflammatory effects of ω-3 PUFAs may be through activation of the AMPK/SIRT1 pathways. To address this hypothesis, we measured cytokine expression, and examined NF-κB signaling in ω-3 PUFA-treated macrophages using luciferase reporter assays, electrophoretic mobility shift assays (EMSA) and Chromatin immunoprecipitation (ChIP) assays. We also examined the effects of ω-3 PUFAs on AMPK expression, phosphorylation and activity, and SIRT1 expression in macrophages. We further tested the ability of ω-3 PUFAs to deacetylate the NF-κB subunit p65 and determined whether SIRT1 is required for ω-3 PUFAs to inhibit NF-κB signaling and its downstream cytokine expression in SIRT1-knockdown macrophages.

## Results

### ω-3 PUFAs suppress LPS-induced cytokine expression in macrophages via antagonizing NF-κB pathway

**Figure 1:**
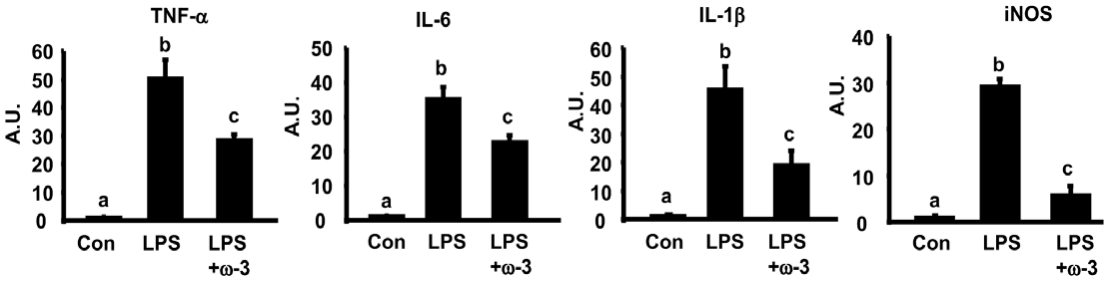
ω-3 PUFAs suppress LPS-induced pro-inflammatory gene expression. Raw264.7 macrophages were pre-treated with ω-3 PUFA mixtures EPA/DHA (50 µM each) for 24 hours and then treated with LPS (100 ng/ml) in the presence or absence of ω-3 PUFA for additional 4 hours. The expression of pro-inflammatory genes was measured by real-time RT-PCR and normalized to cyclophilin. All data are expressed as mean ± SE, n = 6. Statistical significance is indicated by the presence of different superscripts. Groups labeled with the same superscripts are not statistically different from each other. Groups labeled with different superscripts are statistically different from each other. A.U.: Arbitrary Units.

**Figure 2:**
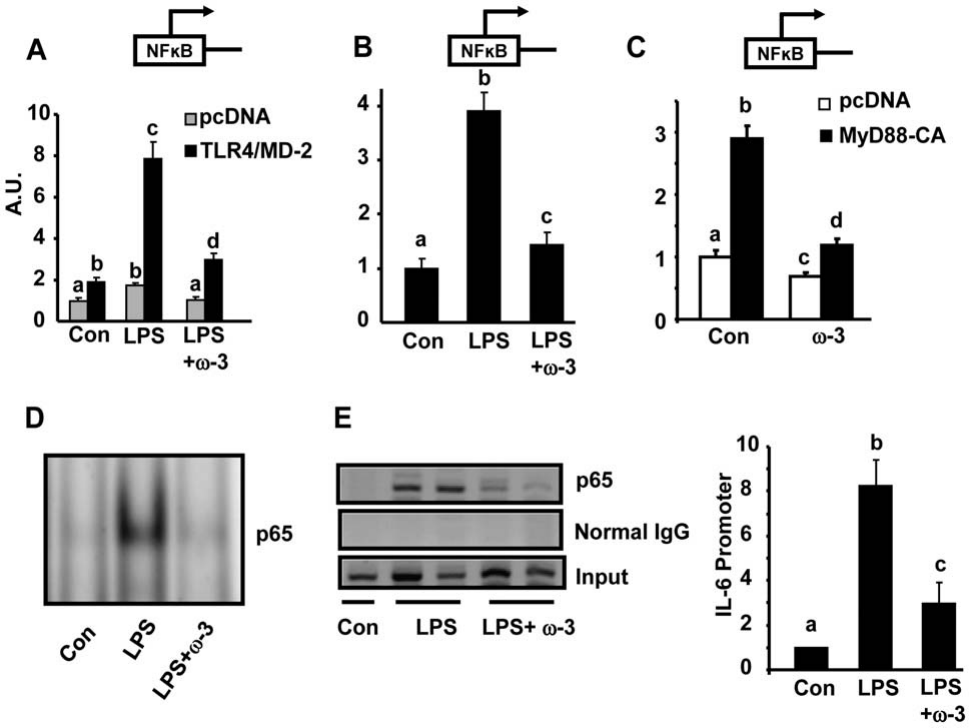
ω-3 PUFAs antagonize NF-κB signaling in macrophages. (A) ω-3 PUFAs suppress TLR4/NF-κB signaling in transfected 293T cells. 293T cells were transfected with TLR4/MD-2 expression vectors, and NF-κB luciferase reporter constructs. Transfected cells were pre-treated with ω-3 PUFA mixtures EPA/DHA (50 µM each) for 24 hours and then stimulated with LPS (100 ng/ml) in the presence or absence of ω-3 PUFA for additional 24 hours. NF-κB luciferase activity was measured using a Dual-Luciferase Reporter Assay. A.U.: Arbitrary Units. (B) ω-3 PUFAs suppress the NF-κB signaling in macrophages. Raw264.7 macrophages were transfected with NF-κB luciferase reporter constructs alone. The treatment is the same as described in (A). (C) ω-3 PUFAs act on the downstream signal(s) of TLR4 to inhibit NF-κB. Raw264.7 macrophages were transfected with CA-MyD88 expression vectors and NF-κB luciferase reporter constructs. (D) ω-3 PUFAs blocks NF-κB DNA binding. EMSA was conducted to examine the NF-κB DNA binding and was conducted as described in Materials and Methods. (E) ω-3 PUFAs blocks the NF-κB subunit p65 binding to the IL-6 promoter. ChIP assays were conducted to examine p65 DNA binding and were conducted as described in Materials and Methods. SYBR Green quantitative PCR was used to quantitate the immunoprecipitated DNA. For (A)–(C), all data are expressed as mean ± SE, n = 6. Groups labeled with the same superscripts are not statistically different from each other. Groups labeled with different superscripts are statistically different from each other.

We first determined the ability of ω-3 PUFAs to antagonize macrophage inflammation. We found that pre-treatment of Raw264.7 macrophages with ω-3 PUFA mixture EPA/DHA (50 µM each) significantly suppressed LPS-induced expression of pro-inflammatory genes including TNF-α, IL-6, IL-1β, and iNOS (**Fig. 1**). This is consistent with the findings we and others have previously reported in macrophages [20], [21], [22]. To explore whether ω-3 PUFAs acts on the NF-κB pathway to antagonize cytokine expression, we established a NF-κB reporter system where 293T cells were transiently transfected with expression vectors for TLR4 and its cofactor MD-2, together with NF-κB luciferase reporter constructs. Cells were then pre-treated with EPA/DHA mixture (50 µM each) overnight and stimulated with LPS (100 ng/ml). **Fig. 2A** shows that ω-3 PUFAs significantly suppressed NF-κB luciferase reporter activity induced by LPS in 293T cells transfected with TLR4 and MD-2 expression vectors. To further determine whether ω-3 PUFAs antagonize the NF-κB signaling in macrophages with endogenous TLR4 and its signaling machinery, Raw264.7 cells were transfected with NF-κB luciferase reporter constructs alone. In consistence, pre-treatment of ω-3 PUFAs inhibited LPS-induced NF-κB luciferase reporter activity in macrophages (**Fig. 2B**). We next determined what step(s) in the TLR4 signaling cascade ω-3 PUFAs act on to antagonize NF-κB. Raw cells were transfected with expression vectors of constitutively active (CA) form of MyD88, an immediate adaptor protein of TLR4. We found that ω-3 PUFAs were still able to inhibit NF-κB reporter activation induced by CA-MyD88 (**Fig. 2C**), suggesting that ω-3 PUFAs likely act on the downstream signal(s) of TLR4 to inhibit NF-κB. We then performed EMSA to further confirm the inhibitory effect of ω-3 PUFAs on endogenous NF-κB signaling in macrophages. As shown in **Fig. 2D**, treatment of Raw cells with ω-3 PUFAs prevented NF-κB DNA binding stimulated by LPS (100 ng/ml). We next performed chromatin immunoprecipitation (ChIP) assays to examine the NF-κB subunit p65 binding to the consensus sequence of the IL-6 promoter. Similarly, ω-3 PUFAs blocked LPS-induced p65 DNA binding to the IL-6 promoter (**Fig. 2E**, left panel). Using SYBR Green PCR to quantitate the immunoprecipitated DNA from the ChIP assays, we further confirmed the inhibitory effects of ω-3 PUFAs on p65 DNA binding to the IL-6 promoter (**Fig. 2E**, right panel). These data suggest that the anti-inflammatory effect of ω-3 PUFAs is mainly mediated via inactivation of NF-κB signaling.

### ω-3 PUFAs activate AMPK and enhances SIRT1 expression in macrophages

**Figure 3:**
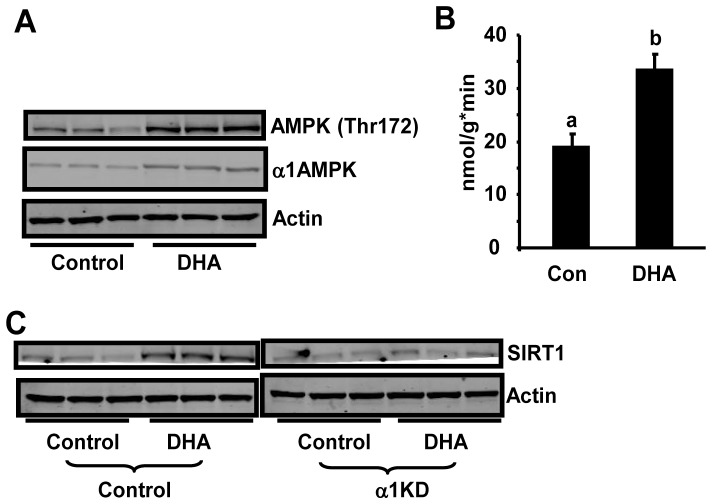
ω-3 PUFAs activate AMPK and enhances SIRT1 expression in macrophages. (A) ω-3 PUFAs increasesα1AMPK protein levels and phosphorylation. Raw264.7 macrophages were treated with DHA (100 µM) for 24 hours. α1AMPK protein and AMPK phosphorylation (Thr172) was measured by immunoblotting with specific antibodies. (B) ω-3 PUFAs increase α1AMPK activity. Raw264.7 macrophages were treated with DHA (100 µM) for 24 hours. α1AMPK activity was measured using an immunocomplex assay with SAMS peptide as described in Materials and Methods. Data are expressed as mean ± SE, n = 6. Groups labeled with the same superscripts are not statistically different from each other. Groups labeled with different superscripts are statistically different from each other. (C) DHA enhances SIRT1 protein levels in control but not in α1AMPK knockdown cells. Macrophages with α1AMPK knockdown were treated with DHA (100 µM) for 24 hours. SIRT1 protein was measured by immunoblotting with specific antibody.

We previously found that two nutrient sensors AMP-activated protein kinase (AMPK) and SIRT1 interact to regulate macrophage inflammation [19]. Therefore, we determined whether ω-3 PUFAs antagonize macrophage inflammation via activation of AMPK/SIRT1 pathways. We found that treatment of Raw264.7 macrophages with the ω-3 PUFA DHA for 24 hours significantly stimulated AMPK phosphorylation and α1AMPK activity (**Fig. 3A and 3B**). DHA treatment also increased α1AMPK protein levels (**Fig. 3A**), which may contribute to the increased AMPK phosphorylation and activity. AMPK and SIRT1 show striking similarities in sensing nutrient supply and regulating metabolic pathways and are likely to interact to perform these functions. We previously demonstrated that activation of AMPK increases SIRT1 expression in macrophages [19]. Here we determined whether activation of AMPK signaling by ω-3 PUFAs increases SIRT1 expression. Using macrophages with α1AMPK knockdown by lentiviral ShRNA [19], we found that DHA treatment significantly enhanced SIRT1 protein levels, while DHA was unable to do so in α1AMPK knockdown cells (**Fig. 3C**), suggesting that the ability of ω-3 PUFAs to stimulate SIRT1 expression requires AMPK.

### SIRT1 is required for ω-3 PUFAs to deacetylate NF-κB and antagonize its signaling in macrophages

**Figure 4:**
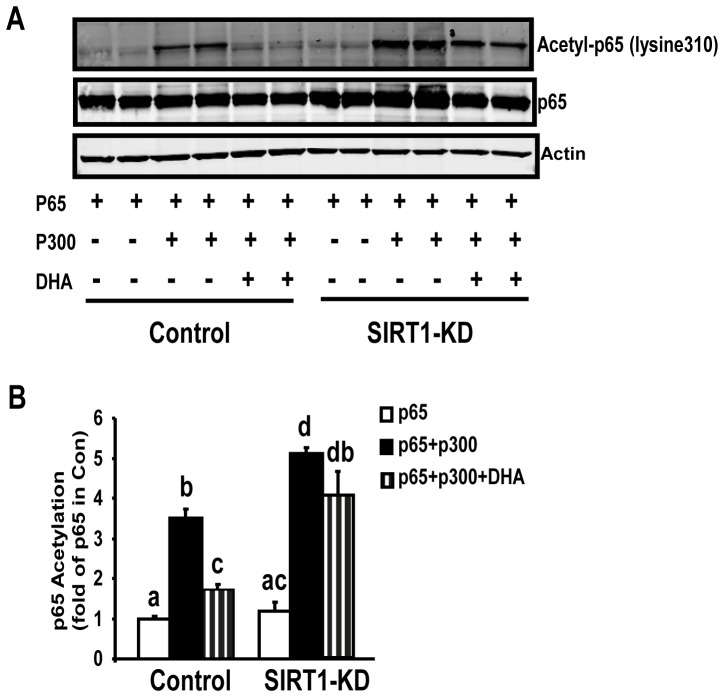
SIRT1 knockdown reduces the ability of ω-3 PUFAs to deacetylate NF-κB in macrophages. A representative blot was shown in (A), and the blots were quantitated with a Li-COR Odyssey Infrared System (B). The SIRT1 knockdown or control macrophages were transfected with expression vectors for p65 or p300, and were then treated with DHA (100 µM) for 24 hours. Acetylation of p65 at lysine310 and SIRT1 protein were measured by immunoblotting with specific antibody. Groups labeled with the same superscripts are not statistically different from each other. Groups labeled with different superscripts are statistically different from each other.

**Figure 5:**
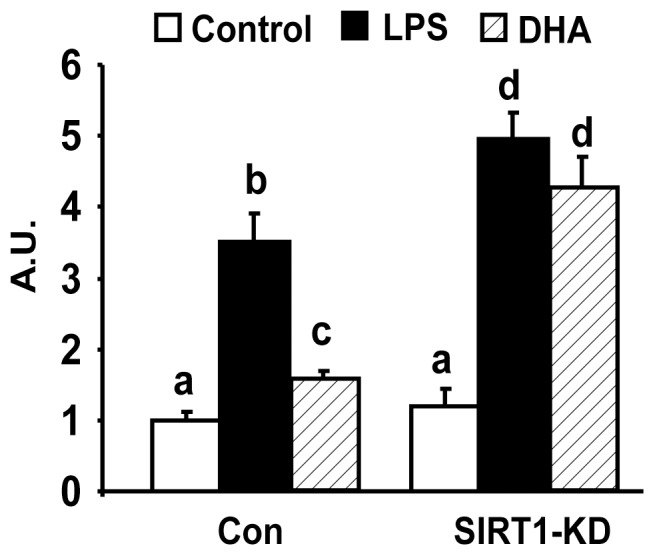
SIRT1 knockdown reduces the ability of ω-3 PUFAs to antagonize NF-κB signaling in macrophages. The SIRT1 knockdown or control macrophages were transfected with NF-κB luciferase reporter constructs. Transfected cells were pre-treated with DHA (100 µM) for 24 hours and then stimulated with LPS (100 ng/ml) in the presence or absence of ω-3 PUFA for additional 24 hours. NF-κB luciferase activity was measured using a Dual-Luciferase Reporter Assay. Data are expressed as mean ± SE, n = 6. Groups labeled with the same superscripts are not statistically different from each other. Groups labeled with different superscripts are statistically different from each other. A.U.: Arbitrary Units.

**Figure 6:**
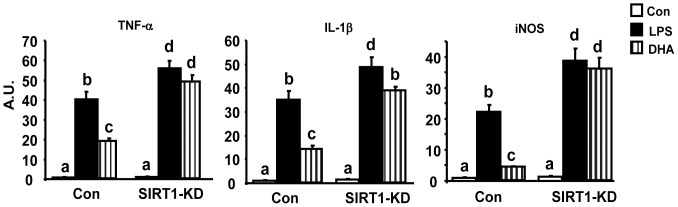
SIRT1 is required for ω-3 PUFAs to suppress LPS-induced expression of pro-inflammatory genes in macrophages. The SIRT1 knockdown or control macrophages were treated with DHA (100 µM) for 24 hours and then treated with LPS (100 ng/ml) in the presence or absence of ω-3 PUFA for additional 4 hours. The expression of pro-inflammatory genes was measured by real-time RT-PCR and normalized to cyclophilin. All data are expressed as mean ± SE, n = 6. Statistical significance is indicated by the presence of different superscripts. Groups labeled with the same superscripts are not statistically different from each other. Groups labeled with different superscripts are statistically different from each other. A.U.: Arbitrary Units.

We have previously shown that SIRT1 antagonizes NF-κB (p65) activity by deacetylating its lysine 310 in macrophages [19]. We tested whether ω-3 PUFAs are also capable of deacetylating NF-κB in macrophages, which requires SIRT1. Using macrophages with SIRT1 knockdown by lentiviral ShRNA [19], we found that in control cells, treatment of the ω-3 PUFA DHA substantially blocked acetylation of p65 at lysine310 induced by p300 (**Fig. 4A**), an acetyltransferase widely used to acetylate p65 [19]. Quantitation of the blot showed that p300 transfection stimulated a 3.5-fold increase of p65 acetylation, while DHA treatment significantly blocked this stimulation by more than 50% (p<0.05, **Fig. 4B**). In contrast, DHA failed to fully deacetylate p65 at lysine310 in SIRT1 knockdown cells (**Fig. 4A**). In SIRT1 knockdown cells, p300 transfection markedly increased p65 acetylation by 5.1 folds. However, DHA treatment failed to prevent the stimulation of p65 acetylation by p300 in knockdown cells (p = 0.2, **Fig. 4B**). We further determined whether SIRT1 is required for ω-3 PUFAs to antagonize NF-κB signaling. DHA treatment substantially blocked LPS-stimulated NF-κB reporter activity in control macrophages, whereas DHA failed to exert the same action in SIRT1 knockdown macrophages (**Fig. 5**). We finally measured the downstream target genes of NF-κB. In parallel, DHA significantly suppressed LPS-induced expression of pro-inflammatory genes including TNF-α, IL-1β, and iNOS, in control cells, but not in SIRT1-knockdown cells (**Fig. 6**). Therefore, these data indicate that SIRT1 mediates the anti-inflammatory effects of ω-3 PUFAs in macrophages.

## Discussion

This study was designed to test the hypothesis that ω-3 PUFAs antagonize macrophage inflammation through activation of AMPK/SIRT1 pathways. The plausibility of this hypothesis was driven by several prior findings on ω-3 PUFAs' anti-inflammatory effects and AMPK and SIRT1 as novel cellular mediators linking nutrient metabolism and inflammation. First, we and others have previously shown that ω-3 PUFAs antagonize macrophage inflammation [20], [21], [22]. However, the cellular mechanisms underlying the anti-inflammatory effects of ω-3 PUFAs are not completely understood. Second, we recently demonstrated that two nutrient sensors AMPK and SIRT1 negatively regulate macrophage inflammation [19]. It would be interesting to know whether these two nutrient sensors also respond to ω-3 PUFAs, a group of beneficial nutrients commonly seen in supplementation (fish oil) and dietary sources (e.g. fish), and mediate their anti-inflammatory effects. Indeed, we found that ω-3 PUFAs activate AMPK and SIRT1 pathways that in turn deacetylate the NF-κB subunit p65 and down-regulate its signaling, leading to suppression of pro-inflammatory gene expression.

The most significant finding in our study is that the anti-inflammatory effect of ω-3 PUFAs may be mediated through activation of AMPK/SIRT1 pathways, which results in down-regulation of NF-κB signaling by deacetylating its subunit p65. We previously demonstrated that AMPK and SIRT1 can detect excess nutrients in diet-induced obesity and serve as negative regulators of nutrient stress-induced inflammation [19]. We found that AMPK signaling in adipose tissue and macrophages are substantially down-regulated by inflammatory stimuli LPS and in diet-induced obesity [19]. To test whether the down-regulation of AMPK signaling might be physiologically significant and contributes to obesity-induced inflammation, we explored the role of AMPK in regulation of macrophage inflammation in both gain- and loss- of function studies. We showed that AMPK activates SIRT1 to suppress macrophage inflammation [19]. The underlying mechanism includes the ability of AMPK and SIRT to deacetylate NF-κB, whose acetylation status affect NF-κB activity and signaling [23]. Based on these observations, we determined: 1) whether AMPK/SIRT1 not only detects excess unhealthy nutrients (e.g. saturated lipids) associated with obesity, but also responds to healthy nutrients (e.g. ω-3 PUFAs) beneficial for prevention and treatment of obesity-associated metabolic disorders; and 2) whether activation of AMPK/SIRT1 in response to ω-3 PUFAs antagonizes macrophage inflammation via antagonism of NF-κB signaling by deacetylating p65. We found that ω-3 PUFAs increase expression, phosphorylation and activity of the major isoform α1AMPK in macrophages, which further leads to SIRT1 over-expression. Our data suggest that AMPK indeed responds to ω-3 PUFAs. It is noteworthy that other anti-inflammatory/anti-oxidants such as polyphenols can also activate AMPK [24]. It appears that both inflammatory and anti-inflammatory signals converge on AMPK that in turn exerts its actions to regulate inflammation. To study the consequence of AMPK/SIRT1 activation by ω-3 PUFAs, we examined the NF-κB acetylation and signaling. We found that the ω-3 PUFA DHA mimics the effect of SIRT1 to deacetylate NF-κB, and SIRT1 mediates the effect of DHA in deacetylation of NF-κB and inhibition of its signaling. ω-3 PUFAs' anti-inflammatory functions have been extensively investigated and a number of potential mechanisms have been proposed. For instance, ω-3 PUFAs can competitively inhibit the conversion of arachidonate to pro-inflammatory lipid intermediates [16], [17]. ω-3 PUFAs have also been shown to serve as endogenous ligands for PPARγ [18], a known signal that has anti-inflammatory function. Serhan's and his colleagues have also identified the anti-inflammatory lipid mediators such as resolvins and protectins that mediate ω-3 PUFAs' effects [13], [14], [25]. More recently, Olefsky's group has reported a novel G-protein coupled receptor GPR120 that mediates the potent anti-inflammatory actions and insulin sensitizing effects of ω-3 PUFA. It is not clear how ω-3 PUFA regulation of AMPK/SIRT1 would fit and interact with the other pathways to regulate inflammation. It is possible that these pathways may be intertwined. For example, as a ligand, ω-3 PUFAs can activate PPARγ that has been shown to activate AMPK [26], [27]. It is also noteworthy that although we demonstrate that anti-inflammatory effect of ω-3 PUFAs is mediated through antagonism of NF-κB signaling, we do not exclude the possibility that ω-3 PUFAs may also act on other inflammatory pathways such as JNK and iKK, which appears to be down-regulated by ω-3 PUFAs in previous reports [9], [15]. It is conceivable that all these pathways may not be mutually exclusive, and probably have crosstalk.

In summary, we first demonstrate that ω-3 PUFAs suppress LPS-induced cytokine expression in macrophages. The anti-inflammatory effect of ω-3 PUFAs is likely mediated through antagonism of NF-κB signaling. We then demonstrate that AMPK/SIRT1 pathways are downstream signals that mediate ω-3 PUFAs' anti-inflammatory effects. ω-3 PUFAs activates AMPK signaling by increasing its protein levels, which further leads to increased SIRT1 protein expression. More importantly, DHA mimics the effect of SIRT1 on deacetylation of NF-κB, and the full capacity of DHA to deacetylate NF-κB and inhibit its signaling and downstream cytokine expression requires SIRT1. We conclude that ω-3 PUFAs negatively regulate macrophage inflammation by deacetylating NF-κB, which acts through activation of AMPK/SIRT1 pathway. AMPK and SIRT1, two classic energy sensors that play key roles in regulating energy metabolism, may serve as novel cellular mediators for the anti-inflammatory effects of ω-3 PUFAs.

## Materials and Methods

### Antibodies and Reagents

Phospho-AMPK (Thr172) and acetyl-p65 (lysine-310) were purchased from Cell Signaling (Beverly, MA). Rabbit polyclonal antibodies against SIRT1 and α1AMPK were obtained from Upstate (Lake Placid, NY). Rabbit polyclonal antibodies against p65 and goat polyclonal antibodies against actin were from Santa Cruz (Santa Cruz, CA). EPA and DHA were purchased from Sigma-Aldrich (St. Louis, MO).

### Cell culture

Raw264.7 macrophages were purchased from American Type Culture Collection (ATCC, Manassas, VA) and cultured in Dulbecco's Modified Eagle's Medium (DMEM) containing 10% heat-inactivated FBS.

### AMPK activity

Cell lysates (50 µg) were immunoprecipitated with specific antibodies (Upstate) against the α1 subunit bound to protein-G sepharose beads. The kinase activity of the immunoprecipitates was measured using “SAMS” peptide and [γ-^32^P]ATP.

### Plasmid constructs and transfection

Murine TLR4, MD-2, and MyD88-CA expression vectors were described previously [20]. Raw264.7 macrophages or 293T cells were transfected with expression vectors using a SuperFect Transfection Reagent kit (Qiagen, Valencia, CA).

### Luciferase reporter assay

pNFκB-Luc and pRL-SV40 vectors were purchased from BD Biosciences-Clontech (Mountain View, CA). Luciferase activity was measured using a Dual-Luciferase Reporter Assay kit (Promega, Madison, WI).

### Lentiviral ShRNA knockdown

SIRT1 and α1AMPK lentiviral ShRNA knockdown were conducted as we previously described [19]. The SIRT1 and α1AMPK lentiviral ShRNA vectors were purchased from Open Biosystems (Huntsville, AL). The ShRNA lentivirus was generated according to the instructions. Briefly, the ShRNA or control lentiviral vectors were co-transfected with the packaging plasmid (pCMV-dR8.91, the Broad Institute, Cambridge, MA) and the envelope plasmid (VSV-G, the Broad Institute) into 293T cells. Medium containing the lentivirus was harvested and filtered, and was used to infect Raw264.7 cells. The infected cells were selected with puromycin (8 µg/ml) for 8 days, and the surviving cells were pooled and used for experiments.

### Chromatin immunoprecipitation (ChIP) assay

ChIP was conducted using a ChIP assay kit (Upstate) as we previously described [19]. Briefly, cells were fixed with 1% of formaldehyde and then harvested in cell lysis buffer (5 mM PIPES, 85 mM KCl, and 0.5% NP-40, supplemented with protease inhibitors, pH 8.0). The lysates were sonicated to shear genomic DNA to an average fragment length of 200–1000 bp. Lysates were centrifuged, and the supernatants were collected. The supernatants underwent overnight immunoprecipitation, elution, reverse cross-link, and protease K digestion. A mock immunoprecipitation with normal serum IgG was also included as a negative control for each sample. The DNA recovered from phenol/chloroform extraction was used for SYBR Green quantitative PCR (Stratagene, Santa Clara, CA), and the DNA quantitation value of each sample was further normalized with the DNA quantitation of individual input control.

### Electrophoretic mobility shift assays (EMSA)

EMSA was conducted as we previously described [20]. The consensus NF-κB oligonucleotides (Promega, Madison, WI) were end-labeled with [γ-^32^P]-ATP (Perkin Elmer, Boston, MA) using T4 polynucleotide kinase (Promega). The protein-DNA complexes were resolved on a Novex 6% DNA retardation gel (Invitrogen, Carlsbad, CA). Gels were dried and analyzed by a phosphor-imaging system (Molecular Dynamics, Sunnyvale, CA).

### Total RNA extraction and quantitative RT-PCR

Macrophage total RNA was extracted using the Tri Reagent kit (Molecular Research Center, Cincinnati, OH), according to manufacturer's protocol. The expression of genes of interest was assessed by quantitative RT-PCR (ABI Universal PCR Master Mix, Applied Biosystems, Foster City, CA) using a Stratagene Mx3000p thermocycler (Stratagene, La Jolla, CA), as we previously described [20]. The primer and probe pairs used in the assays were purchased from Applied Biosystems.

### Immunoblotting

Immunoblotting was conducted as we previously described [19]. Briefly, the transferred membranes were blocked, washed, incubated with various primary antibodies overnight at 4°C, and followed by Alexa Fluor 680-conjugated secondary antibodies (Invitrogen) at room temperature for 2 hrs. The blots were developed with a Li-COR Odyssey Infrared Imager system (Li-COR Biosciences, Lincoln, NE).

### Statistics

All data are expressed as mean±SEM. Data were evaluated for statistical significance by one way ANOVA, and statistical significance for comparison of means of different groups was calculated by the least-significant-difference test using the SPSS software package version 11.5. p<0.05 was considered significant.
